# The Time is Now, but Mind the Gaps: Communities – Governance – Implementation

**DOI:** 10.5334/ijic.7687

**Published:** 2023-07-05

**Authors:** Mirella M. N. Minkman

**Affiliations:** 1 Tilburg University-TIAS Businessschool, CEO Vilans, National Center of Expertise for care and support, The Netherlands

**Keywords:** integrated care governance, linking communities, implementation

## Can we close the gaps?

Supported by the increasing body of knowledge on how to achieve coherent, integrated care and support in communities and countries, the movement towards integrated care is now stronger than ever. The current stress on many healthcare systems increases the urgency. International bodies have pointed out the need to focus on person- and community-centred integrated care. The WHO frameworks, knowledge collected by the International Foundation of Integrated care, plus the abundance of practice and research experiences worldwide, highlight the vital elements, preconditions and lessons learned. Although contexts differ and must be taken into account, it is no longer arguable that elements such as enhanced coordination, goal driven and domain overarching collaboration and supportive governance structures need to be implemented [1]. However, the gaps between what is and what could be remains. For decades, the London Underground has been warning us to ‘mind the gap’ when disembarking from the train onto the platform. It is a simple but effective intervention that keeps everyone alert and helps to get everybody on board safely. How to mind the gaps in integrated care?

## Bridging the gaps

There are many gaps, and to bridge them all it helps to begin by briefly discussing three of the gaps you might cross.

1. The awareness that integrated care is not about ‘connecting existing services’ but about supporting healthy living. The scarcity of resources in many countries (staff, means or energy) speeds up the awareness that the community and citizens themselves – not only the care workforce and facilities – are the most important change facilitators. At the same time, it is also the largest group and therefore not easy to engage. It requires other behaviours and other roles on both sides.
2. The necessary innovation in governance and decision-making structures is an intensive but important gap to bridge. Governance of integrated care, defined as the necessary collaborative leadership, and suitable approaches of accountability, supervision and financial mechanisms, almost directly affects shifts in power balance. Power balance is complex to change.
3. The third gap is implementation. The gap between knowing and doing or being able to act is often huge. Besides combined bottom-up and top-down implementation approaches, the translation of knowledge into practical behavioral change and supportive learning environments is a challenge.

## The Communities and workforce gap

The first gap is involving communities, citizens or ‘patients’ as partners in care. For decades, terms like client or patient-centered are used, however, the professional and organisational perspective still often takes the lead. A diversity of articles in IJIC’s Special Issue about people-driven care, concluded that the state of the evidence is still limited but emerges on the issue that people driven services are better coordinated and serve people’s needs [2]. The intention to involve citizens is often present, however imbalances in knowledge and power, also related to culture or beliefs, has so far influenced the success in some countries. Especially in settings where ‘normal living’ (elderly care or care for people with disabilities) instead of a more short-term treatment experience is dominant, a closer relationship between citizens, families and communities is important. Connecting formal and informal care is key for healthy and caring societies. At the same time both sides experience difficulties in connecting with each other and are subject to change themselves. Boards of care facilities often raise the question how to shift the focus of their staff towards collaboration with families and the community, when they are educated to care for people and *take over*. It is their profession and often also their passion to care. Involving families and discussing what they can do for their loved one is not easy. Besides the expectation that staff should care for their family member, relatives themselves often argue about what is most appropriate. Concepts like reablement support the movement towards including citizens and families by strengthening their own capacities [3]. The reablement concept gains evidence from positive effects and more widespread implementation. In some Scandinavian countries like Norway, reablement has already been introduced in 75% of the municipalities. In other countries like the Netherlands, more group or community-based initiatives of ‘caring for each other’ are on the rise. These citizen initiatives include social activities, supporting each other with daily tasks like grocery shopping, a ride to the hospital or even include a new ‘profession’ like the ‘municipality supporter’ [4]. Knowledge about these community-based approaches and change in ‘the workforce’ is an important area for further research. Also, because internationally, the definitions of informal and formal care, inside or outside the labour market, private or public, are ambiguous. The ‘zorgzame buurten’ concept in Belgium (caring communities) consists of three pillars including domain overarching collaboration towards integrated care and services. Connecting formal and informal care is another pillar [5]. This is where the gap becomes clear. There is the community approach that seeks connection with formal care and the formal care facilities that need to strengthen their connections with society. Glimmerveen [6] interestingly found in his empirical research, that although citizen participation aims to blur the lines between citizens and professional workers, these boundaries often then become the subject of tough discussions. Internal power balances within healthcare settings define which topics citizens are allowed to speak and participate in. Also, informal carers involved in healthcare settings are dependent on the legitimacy of their position – or the absence of it. In practice this can lead to defining ‘constructive participation’ as the norm, whereas citizens who act as ‘critical opposites’ are excluded. Without the ‘top’ support within organisations, their role in decision making stays marginal [6]. Besides developing a diversity of connections between formal and informal care, understanding the role of rebalancing powers is also important to bridge the gap.

## The power and governance gap

As described in previous publications, traditional governance within organisations does not match the governance needed in collaborative arrangements [7,8]. Models based on network governance that are more horizontal, non-hierarchic, based on shared responsibilities and trust as basic values, seem to be more fitting. Working with those models however, requires re-thinking the roles and processes of boards and supervisors. Also, it asks for re-thinking governance mechanisms for decision making when there is no formal hierarchy between partners, but dependencies, inequalities and powers that exist [9]. Integrated care governance should also reshape traditional accountability mechanisms. Accountability goes beyond being responsible towards ‘who can pay or who can punish’ (health insurers or inspectorates) but also towards the society or citizens, especially when they are increasingly becoming partners and co-producers of (informal) care as discussed in the first gap. When the role of citizens themselves changes, community or collaborative governance is the new landscape. However, as described by Stone [10], a call to restructure is always a bid to reallocate power. There are always attempts by someone who is not winning in the arena, to shift decision making to an arena where they might prevail. Whereas collaboration in integrated care settings is aimed at a public interest, new configurations of decision making might enable another interest to become dominant. Like in politics, three strategies for shifting powers seem recognisable in integrated care collaborations:

1. Who is given the right to make decisions, who’s voice counts? Changing the composition of the decision making body.
2. Who is allied with whom? What new hierarchies are created? What is the size and shape of the collaboration?
3. Options are shifting the scale and locus of the decision making between local, regional or national. On what scale are collaborations and powers allocated?

Transforming from organisation based governance models towards hybrid decision making and shifting powers, are still often complex gaps to bridge. Connecting resources and also financing mechanisms to the shared goals are crucial to influence decision making and change behavior. Rewarding the desired behavior is a critical (financial) incentive for decision making in the common good. Van der Weert et al [11] concluded that hardly any empirical research is available on the effects of different network structures and governance models on healthcare network performance at different levels or scales. Closing this gap asks for a close interaction between research and practice development, and stakeholders at system level in a country.

## The implementation gap

As we have learned in integrated care history, implementation is not a simple step-by-step fix but must be addressed from a wider spectrum of responses and levels to deal with the ‘wicked problem’. We have learned a lot about fundamental components for implementation [12,13,14]. For example, the importance of building relationships in alliances and networks and the underlying norms and values that matter. Values become visible in behavior and choices of people and play a role in building trust, the (un)willingness to collaborate and the bravery to let go of siloed ways of working.

Furthermore, we know that making intelligent choices about the re-organisation of scarce resources is often needed but takes courage, a rethinking of scale, and a reframing of the organisation of health and social care at every level. There are new horizons in what can be done by communities themselves. Overall, the multi-layered context of integrated care implies the need for intensive learning approaches and learning loops within and also between organisations. This makes the implementation of integrated care services a long-term achievement which should be based on strong fundamentals. We can only accelerate in the long run if we slow down to build these essential fundamentals like adaptive governance mechanisms on every scale [15].

Besides the gap between the necessary timespans and the wish for short term results, implementation also needs knowledge or examples that can be understood and applied. Scientific articles need to be translated into practical suggestions and practical pilots into conceptual knowledge to apply in another context. For implementation ‘snackable knowledge’ needs to be present. Furthermore, facilitated exchange of knowledge and lived experiences, for instance by large scale improvement programmes can help to accelerate and use knowledge more efficiently. Lastly, tables where experienced barriers to implementation (like hindering policies, financial mechanisms, or non-aligned supervision) are resolved will help to escape the pilot phase.

## Conclusion

Like in the London Underground, minding the gaps matters. This volume of our Journal adds knowledge about other possible gaps, how others have made a step, what they have learned, and what it can mean to you. Only if we share and care, keep focused on the aims of healthier and happy lives, can we keep on stepping on and off the platforms safely and keep the trains running. However, there is no learning without falling. Therefore publications about what didn’t succeed or hasn’t worked are also valuable. Let’s mind the gaps, work on them, but never be afraid of them.
